# Revisiting immune checkpoint inhibitors: new strategies to enhance efficacy and reduce toxicity

**DOI:** 10.3389/fimmu.2024.1490129

**Published:** 2024-12-10

**Authors:** Dianying Zhang, Jingjing Zhao, Yujing Zhang, Hongfei Jiang, Dan Liu

**Affiliations:** ^1^ Medical Education Department, Guangdong Provincial People’s Hospital, Zhuhai Hospital (Jinwan Central Hospital of Zhuhai), Zhuhai, China; ^2^ Sleep Medicine Center, Huai’an No.3 People’s Hospital, Huai’an, China; ^3^ Huaian Second Clinical College of Xuzhou Medical University, Huaian, China; ^4^ The Affiliated Cardiovascular Hospital of Qingdao University, Qingdao University, Qingdao, Shandong, China; ^5^ The Affiliated Hospital of Qingdao University, Qingdao University, Qingdao, Shandong, China

**Keywords:** immune checkpoint inhibitors, cancer therapy, biomarker-guided therapy, combination therapies, immune-related adverse events (irAEs)

## 1 Introduction

### 1.1 Background on immune checkpoint inhibitors

In recent years, ICIs have transformed cancer treatment by harnessing the body’s immune system to target and destroy cancer cells (1–3). ICIs work by blocking inhibitory signals that prevent T cells from attacking tumors, thereby reactivating the immune response against cancer. The most common targets for these therapies are the PD-1/PD-L1 and CTLA-4 pathways, which are critical in regulating immune responses (4). For example, nivolumab, a PD - 1 inhibitor, has been a breakthrough in the treatment of melanoma. In a large - scale clinical trial involving patients with advanced melanoma, nivolumab treatment led to a significant improvement in overall survival, with approximately 40% of patients surviving for more than five years compared to less than 20% with traditional chemotherapy (5). Pembrolizumab, another PD - 1 inhibitor, has shown remarkable efficacy in non - small cell lung cancer (NSCLC). In a phase III trial, it demonstrated an objective response rate of around 20% - 30% in previously treated patients and has since been incorporated into first - line treatment regimens, improving survival outcomes and quality of life for many patients (6). Ipilimumab, a CTLA - 4 inhibitor, has had a transformative impact on metastatic melanoma. It was the first drug to show an overall survival benefit in this difficult - to - treat cancer, increasing the median survival time by several months and providing a new treatment option for patients with limited alternatives (7, 8). These examples clearly illustrate the remarkable success of ICIs in different cancer types and their ability to revolutionize cancer treatment. While ICIs have demonstrated efficacy in a range of cancers, including melanoma and non - small cell lung cancer, their potential in treating bone tumors remains underexplored. Addressing this gap, the article also considers strategies tailored to enhance ICI efficacy specifically in bone tumor cases.

These therapies have been particularly revolutionary for cancers that were previously difficult to treat, offering the potential for long-term remission in some patients. However, despite these successes, ICIs are not universally effective. Many patients do not respond to treatment, and those who do may develop resistance over time (9). Additionally, the activation of the immune system can lead to severe immune-related adverse events (irAEs), which can affect various organs and require careful management (10). The challenges of suboptimal efficacy and significant toxicity highlight the need for refined strategies in the use of ICIs. Personalized approaches, combination therapies, and the development of next-generation ICIs with improved specificity and safety profiles are essential to maximizing the therapeutic potential of these treatments.

### 1.2 Challenges with ICIs

Despite the transformative potential of ICIs, their use is accompanied by significant challenges. One of the foremost issues is the variable response among patients. While some individuals experience dramatic and long-lasting tumor regression, many others do not respond to ICIs at all, a phenomenon known as primary resistance (10). Even among responders, a subset may develop acquired resistance over time, leading to cancer progression after an initial period of remission (11).

Another critical challenge is the occurrence of irAEs. These toxicities arise from the over activation of the immune system and can affect multiple organs, including the skin, gastrointestinal tract, liver, and endocrine system (12). IrAEs range from mild to severe and can be life-threatening, necessitating the use of immunosuppressive treatments that might diminish the anti-tumor efficacy of ICIs (13). Additionally, the phenomenon of resistance is a major hurdle. Primary resistance, where patients do not respond to ICIs from the start, may be attributed to several factors. Tumors with low immunogenicity, due to a lack of tumor - specific antigens or a suppressive tumor microenvironment rich in immunosuppressive cells like regulatory T cells (Tregs) and myeloid - derived suppressor cells (MDSCs), can prevent effective T cell activation and infiltration (14). Genetic alterations within the tumor cells, such as mutations in the interferon - gamma pathway genes, can also lead to primary resistance (15). Acquired resistance, which develops over time in initially responsive patients, may involve the upregulation of alternative immune checkpoint pathways, such as TIM - 3 and LAG - 3, that compensate for the blocked PD - 1/PD - L1 or CTLA - 4 pathways (16). Tumor cells can also adapt by losing expression of target antigens or developing mechanisms to evade immune recognition, such as through antigen - presentation machinery defects (17). Understanding these mechanisms underlying resistance is crucial as it sets the stage for the later discussion on emerging strategies to overcome resistance. Furthermore, the high cost of ICIs presents a significant barrier to access, limiting their availability to a broader patient population (18). These challenges underscore the urgent need for strategies to predict response, manage toxicity, and reduce costs, thereby optimizing the clinical application of ICIs in cancer therapy.

## 2 Current landscape of immune checkpoint inhibitors

### 2.1 Overview of existing ICIs

ICIs targeting the PD-1/PD-L1 and CTLA-4 pathways have become integral to modern cancer therapy (19, 20). PD-1 inhibitors, such as nivolumab and pembrolizumab, prevent the interaction between PD-1 on T cells and its ligand PD-L1 on tumor cells, thereby reinvigorating T cells to attack cancer (21). These inhibitors have shown substantial efficacy in treating various cancers, including melanoma, non-small cell lung cancer (NSCLC), and renal cell carcinoma. Similarly, CTLA-4 inhibitors like ipilimumab enhance T cell activation by blocking the inhibitory signals that dampen immune responses, particularly in the context of metastatic melanoma (22).

Despite these successes, not all patients benefit from these therapies. Response rates vary significantly, with some tumors being more resistant due to various factors, including the tumor microenvironment and genetic mutations (23). Bone tumors, particularly osteosarcoma, present unique challenges in immunotherapy due to their complex microenvironment (24). This article explores how tailored approaches could potentially overcome these barriers, leading to improved outcomes in bone tumor patients. Additionally, while ICIs have transformed the outlook for many patients, they are not curative for all, and a significant portion of patients eventually develop resistance (25). These limitations highlight the need for ongoing research to refine these treatments and develop new strategies to overcome resistance and improve response rates.

### 2.2 Toxicity profile

The introduction of ICIs has marked a significant advancement in cancer therapy, but their use is associated with a distinct set of toxicities known as irAEs. Unlike traditional chemotherapy-induced toxicities, irAEs result from the overactivation of the immune system as it begins to attack not only cancer cells but also healthy tissues (26). These adverse events can affect almost any organ system, with the most commonly impacted being the skin, gastrointestinal tract, liver, and endocrine glands (27).

Dermatologic toxicities, such as rash and pruritus, are among the most frequent irAEs, often appearing early in treatment (28). Gastrointestinal irAEs, including colitis and diarrhea, can range from mild to severe, potentially leading to life-threatening complications if not promptly managed (29). Hepatotoxicity, manifesting as elevated liver enzymes or hepatitis, is another significant concern and requires careful monitoring and sometimes the cessation of ICI therapy (30). Endocrine irAEs, such as thyroiditis, adrenal insufficiency, and hypophysitis, can lead to long-term hormonal imbalances, necessitating ongoing hormone replacement therapy. Pulmonary toxicity, including pneumonitis, is less common but can be severe and life-threatening. Cardiovascular and neurological toxicities, though rare, can also occur and pose serious risks.

The management of irAEs often involves the use of corticosteroids and other immunosuppressants to mitigate the immune response (31). However, this approach can compromise the anti-tumor efficacy of ICIs, creating a delicate balance between controlling toxicity and maintaining therapeutic benefit. The unpredictability and potentially severe nature of irAEs underscore the need for close monitoring, early intervention, and the development of more selective ICIs that minimize off-target effects. As the use of ICIs continues to expand, understanding and managing these toxicities will be crucial for optimizing patient outcomes.

## 3 Emerging strategies to enhance efficacy

### 3.1 Biomarker-guided therapy

Biomarker-guided therapy represents a promising approach to enhancing the efficacy of ICIs by tailoring treatments to the unique characteristics of each patient’s tumor (32). Biomarkers such as PD-L1 expression, tumor mutational burden (TMB), and microsatellite instability (MSI) have been identified as potential predictors of response to ICIs. For example, high PD-L1 expression on tumor cells is often associated with a better response to PD-1/PD-L1 inhibitors, making it a critical factor in patient selection for these therapies. While high PD - L1 expression on tumor cells is often associated with a better response to PD - 1/PD - L1 inhibitors, it is acknowledged that other factors can also influence the efficacy of ICIs. For instance, the presence of immunosuppressive cells within the tumor microenvironment, such as regulatory Tregs and MDSCs, can dampen the immune response despite high PD - L1 expression (33). Additionally, genetic alterations within the tumor cells, like mutations in the interferon - gamma pathway genes, may affect the sensitivity of tumors to ICIs even in the presence of high PD - L1 levels (34). Therefore, a comprehensive evaluation that takes into account multiple factors is essential for accurate patient selection and treatment prediction.

Similarly, a high tumor mutational burden, which reflects the number of mutations within a tumor’s DNA, is correlated with increased neoantigen formation, potentially enhancing the immune system’s ability to recognize and attack the tumor (35). Microsatellite instability, a condition of genetic hypermutability, also serves as a biomarker for response to ICIs, particularly in colorectal cancers (36).

By utilizing these biomarkers, clinicians can more accurately identify patients who are most likely to benefit from ICI therapy, thus improving overall outcomes. Currently, there are ongoing efforts to standardize the assessment of biomarkers. Several professional organizations and research consortia are working towards establishing unified testing methods and criteria for biomarker evaluation. This includes standardizing the assays used to measure PD - L1 expression, TMB, and MSI, as well as defining cut - off values for determining biomarker positivity (37–39). Standardization is crucial as it would enhance the reproducibility and generalizability of biomarker - guided treatment strategies. If different laboratories and clinics use inconsistent methods, it could lead to varying results and inaccurate patient selection. With standardized assessment, the reliability of biomarker - based treatment decisions would improve, allowing for more effective implementation of personalized medicine in the context of ICIs. Furthermore, ongoing research is focused on discovering new biomarkers and refining existing ones, which could lead to even more personalized and effective treatment strategies in the future.

### 3.2 Combination therapies

Combination therapies involving ICIs have emerged as a powerful strategy to enhance cancer treatment efficacy (40). By combining ICIs with other therapeutic modalities, such as chemotherapy, targeted therapy, radiotherapy, or even other ICIs, it is possible to overcome resistance mechanisms and achieve more robust and durable responses (41). The rationale behind these combinations lies in the synergistic effects that can be achieved when different treatments target complementary pathways involved in tumor growth and immune evasion.

For instance, chemotherapy and radiotherapy can induce immunogenic cell death, which increases the release of tumor antigens and enhances the subsequent immune response when paired with ICIs (42). Targeted therapies, such as those inhibiting angiogenesis or specific oncogenic pathways, can modify the tumor microenvironment, making it more susceptible to immune-mediated destruction. The combination of different ICIs, such as PD-1/PD-L1 inhibitors with CTLA-4 inhibitors, can simultaneously block multiple immune checkpoints, potentially leading to a more comprehensive activation of the immune system against the tumor.

Recent clinical trials have demonstrated the success of these combinations in various cancers, showing improved response rates and extended survival compared to monotherapy (43). However, combination therapies also pose challenges, including increased toxicity and the complexity of managing multiple treatments (44). Despite these challenges, the continued exploration of combination strategies holds significant promise for improving outcomes in patients who do not respond adequately to ICIs alone.

### 3.3 Optimizing dosing and scheduling

Optimizing the dosing and scheduling of ICIs is a critical strategy for maximizing their therapeutic efficacy while minimizing associated toxicities. Traditional dosing regimens often involve fixed doses or schedules that may not account for individual patient variability in drug metabolism and immune response (45). Emerging evidence suggests that alternative dosing strategies, such as intermittent dosing or dose reductions, can maintain anti-tumor efficacy while potentially reducing the risk of irAEs. These approaches could allow for better management of toxicities, making ICIs more tolerable for a broader range of patients, including those with comorbidities or lower tolerance for treatment.

In addition to dose optimization, adjusting the timing of ICI administration is also being explored as a way to enhance outcomes. For example, administering ICIs in conjunction with other treatments, such as chemotherapy or radiotherapy, at specific intervals may enhance the immune response by taking advantage of the immunomodulatory effects of these therapies (46). Similarly, the timing of ICI administration in relation to the patient’s circadian rhythms and immune cycles is an area of active research, with the potential to further refine treatment schedules for optimal results. These strategies represent promising avenues for improving the safety and effectiveness of ICI therapy.

## 4 Strategies to reduce toxicity

### 4.1 Selective targeting and engineering of ICIs

Selective targeting and engineering of ICIs represent a promising approach to enhancing the specificity and safety of these therapies (47). Traditional ICIs, while effective, can lead to irAEs due to their broad activation of the immune system. To address this, researchers are developing next-generation ICIs that are designed to more precisely target tumor cells while sparing healthy tissues. One strategy involves engineering ICIs with enhanced affinity for tumor-specific antigens or altered immune checkpoint proteins that are predominantly expressed in the tumor microenvironment. This selective targeting reduces off-target effects and minimizes the risk of irAEs, potentially allowing for higher doses or more frequent administration without increasing toxicity (48).

In addition to improving selectivity, advances in protein engineering are enabling the creation of ICIs with optimized pharmacokinetics and pharmacodynamics (49). These engineered ICIs can be designed to have longer half-lives, greater stability, and more controlled activation, which enhances their efficacy and reduces the need for frequent dosing. Furthermore, bispecific antibodies that simultaneously target two immune checkpoints or combine checkpoint inhibition with other immune-stimulating functions are being explored as a way to increase the therapeutic potency of ICIs. These innovations are paving the way for more effective and safer cancer immunotherapies, offering new hope for patients who may not have benefited from existing treatments.

### 4.2 Immune modulation approaches

Immune modulation approaches aim to manage the irAEs associated with ICIs while preserving their therapeutic efficacy (50). One common strategy involves the use of corticosteroids and other immunosuppressive agents to dampen excessive immune responses that cause irAEs. However, this approach can sometimes blunt the anti-tumor effects of ICIs, creating a delicate balance between managing toxicity and maintaining the desired immune activation. Researchers are exploring alternative immune modulators that can more selectively target the pathways involved in irAEs without compromising the overall immune response against the tumor (51).

In addition to pharmacological interventions, immune modulation can also involve adjusting the timing or combination of ICIs with other therapies to modulate the immune response more effectively. For instance, combining ICIs with agents that promote regulatory T cells (Tregs) or other immune-regulating cells might reduce irAEs by controlling the extent of immune activation (52). These approaches are still in the early stages of research but hold promise for making ICI therapy safer and more tolerable, allowing more patients to benefit from these powerful cancer treatments without the burden of severe side effects.

### 4.3 Patient management and monitoring

Effective patient management and monitoring are critical components of optimizing immune checkpoint inhibitor (ICI) therapy. Given the potential for irAEs to affect multiple organ systems, early detection and intervention are essential to prevent severe complications. Routine monitoring of patients receiving ICIs should include regular assessments of symptoms, laboratory tests, and imaging studies to detect any emerging irAEs (53). Early identification allows for prompt management, which can include dose adjustments, temporary discontinuation of therapy, or the initiation of immunosuppressive treatments to control inflammation.

A multidisciplinary approach is often required to manage the diverse toxicities associated with ICIs. Involvement of specialists, such as endocrinologists, gastroenterologists, and pulmonologists, can help in the targeted management of specific irAEs (54). Additionally, patient education plays a crucial role in management, as patients need to be aware of the potential signs and symptoms of irAEs and the importance of reporting them promptly to their healthcare team. This proactive communication can lead to earlier interventions and better outcomes.

Long-term monitoring is also essential, as some irAEs may develop late in the course of treatment or even after therapy has ended. Continued follow-up ensures that any delayed toxicities are managed appropriately and that the overall health and quality of life of the patient are maintained. By integrating comprehensive monitoring protocols and a proactive management approach, clinicians can maximize the therapeutic benefits of ICIs while minimizing the risks, ultimately leading to improved patient outcomes in cancer therapy.

In this article, we explore various strategies aimed at enhancing the efficacy and reducing the toxicity of ICIs. These strategies, which include biomarker-guided therapy, combination therapies, optimizing dosing and scheduling, selective targeting and engineering of ICIs, immune modulation approaches, and comprehensive patient management, are summarized in **Table 1**. This table provides a concise overview of the key approaches and their intended outcomes, illustrating the potential to improve both the safety and effectiveness of ICIs in cancer therapy.

**Table 1:** Strategies for enhancing efficacy and reducing toxicity of immune checkpoint inhibitors.

Strategy	Approach	Intended Outcome
Enhancing Efficacy	Biomarker-Guided Therapy	Personalized treatment based on PD-L1 expression, TMB, and MSI biomarkers, leading to improved patient selection and outcomes.
	Combination Therapies	Enhanced treatment efficacy through synergistic effects of combining ICIs with chemotherapy, radiotherapy, or other ICIs.
	Optimizing Dosing and Scheduling	Maximizing therapeutic benefits while minimizing toxicities by adjusting dosing regimens and treatment schedules.
Reducing Toxicity	Selective Targeting and Engineering of ICIs	Minimized off-target effects by designing ICIs with higher affinity for tumor-specific antigens, leading to reduced irAEs.
	Immune Modulation Approaches	Balancing immune activation and suppression through the use of corticosteroids, immune modulators, and strategic timing of ICIs.

## 5 Future directions

The future of immune checkpoint inhibitor (ICI) therapy lies in overcoming current limitations and expanding the therapeutic potential of these powerful treatments. One of the most promising avenues is the development of next-generation ICIs that offer enhanced selectivity and reduced toxicity (55). Advances in biotechnology are paving the way for engineered antibodies, bispecific molecules, and novel immune checkpoint targets that could provide more effective and safer cancer treatments. These innovations have the potential to broaden the applicability of ICIs to a wider range of cancers, including those that are currently resistant to existing therapies.

Importantly, the development of more effective and targeted ICIs may have a positive impact on the cost - effectiveness of ICI therapy. For example, if next - generation ICIs with higher response rates can be developed, it may reduce the need for multiple lines of treatment or combination therapies that are often costlier. Additionally, improved biomarkers for patient selection could ensure that ICIs are prescribed to those who are most likely to benefit, thereby avoiding unnecessary treatment costs for non - responders. In addition to new drug development, ongoing research is focused on better understanding the mechanisms of resistance to ICIs (56). By identifying the genetic and molecular factors that contribute to primary and acquired resistance, researchers can develop combination strategies that target these pathways and restore sensitivity to ICIs. Furthermore, the integration of biomarkers into clinical practice will allow for more personalized treatment approaches, ensuring that patients receive therapies most likely to be effective based on their individual tumor characteristics.

Another key direction involves refining the timing and sequencing of ICIs in combination with other treatment modalities, such as chemotherapy, radiotherapy, and targeted therapies. Optimizing these combinations can enhance therapeutic outcomes while minimizing adverse effects. As research continues to evolve, there is also growing interest in exploring ICIs in non-cancer indications, potentially opening new frontiers in the treatment of autoimmune diseases and chronic infections. Together, these efforts promise to shape the future of cancer therapy, making ICIs a cornerstone of precision oncology.

## 6 Conclusion

### 6.1 Summary of key points

In summary, ICIs have revolutionized cancer therapy, offering significant benefits but also presenting challenges such as variability in patient response and the risk of irAEs. Advances in biomarker-guided therapy, combination strategies, and the engineering of next-generation ICIs hold promise for overcoming these limitations. Ongoing research into optimizing dosing, patient management, and resistance mechanisms is crucial for enhancing the efficacy and safety of ICIs. By refining these strategies, ICIs can be more effectively integrated into personalized cancer treatment, improving outcomes for a broader range of patients.

### 6.2 Call to action

The continued success of ICIs in cancer therapy hinges on the collaborative efforts of researchers, clinicians, and industry leaders. To fully realize the potential of ICIs, it is essential to prioritize research into understanding and overcoming resistance mechanisms, developing more precise biomarkers, and engineering next-generation ICIs with improved safety profiles. Clinicians must adopt a multidisciplinary approach to patient management, ensuring early detection and prompt intervention for irAEs. Additionally, there is a need for ongoing education and training to help healthcare professionals stay informed about the latest advancements in ICI therapy. By fostering innovation, collaboration, and education, the oncology community can enhance the effectiveness of ICIs, making these therapies more accessible and beneficial to a wider range of patients. The time to act is now, as the ongoing refinement of ICI strategies will be crucial in shaping the future of cancer treatment.

### 6.3 Implications for future cancer therapy

The advancements and refinements in immune checkpoint inhibitor (ICI) therapy are poised to significantly impact the future of cancer treatment. As we continue to develop more precise and personalized approaches, ICIs are likely to become integral components of combination therapies that target multiple aspects of tumor biology. The ongoing research into biomarkers and next-generation ICIs promises to expand their applicability to a broader range of cancers, including those previously resistant to treatment. Additionally, the improved management of irAEs will enable more patients to safely benefit from these therapies. These developments not only enhance the effectiveness of cancer treatment but also pave the way for new therapeutic paradigms that focus on long-term disease control and potentially curative outcomes. The future of cancer therapy will increasingly rely on the integration of ICIs into comprehensive, patient-specific treatment strategies that offer hope for better survival and quality of life.

## References

[1] Qian X Hu W Yan J. Nano-Chemotherapy synergize with immune checkpoint inhibitor- A better option? Front Immunol. (2022) 13:963533. 36016946 10.3389/fimmu.2022.963533 PMC9395615

[2] Liu Z Zhu Y Xie H Zou Z. Immune-mediated hepatitis induced by immune checkpoint inhibitors: Current updates and future perspectives. Front Pharmacol. (2023) 13:1077468. doi: 10.3389/fphar.2022.1077468 36699050 PMC9868416

[3] Xie Q Zhang P Wang Y Mei W Zeng C. Overcoming resistance to immune checkpoint inhibitors in hepatocellular carcinoma: Challenges and opportunities. Front Oncol. (2022) 12:958720. doi: 10.3389/fonc.2022.958720 36119533 PMC9478417

[4] Papouin B Mussini C De Martin E Guettier C. Effets secondaires digestifs et hépatiques des inhibiteurs du checkpoint immunitaire (Immune checkpoint inhibitors: anti-CTLA-4 et anti-PD-1/PD-L1): aspects anatomocliniques. Annales Pathologie. (2018) 38:338–51. doi: 10.1016/j.annpat.2018.07.005 30143243

[5] Kato K Mizuno T Koseki T Ito Y Takahashi K Tsuboi N. Frequency of immune checkpoint inhibitor-induced vasculitides: an observational study using data from the Japanese adverse drug event report database. Front Pharmacol. (2022) 13:803706. doi: 10.3389/fphar.2022.803706 35401222 PMC8992371

[6] Kwok G Yau TCC Chiu JW Tse E Kwong Y-L. Pembrolizumab (Keytruda). Hum Vaccines Immunotherapeutics. (2016) 12:2777–89. doi: 10.1080/21645515.2016.1199310 PMC5137544 27398650

[7] Bestvina CM Pointer KB Karrison T Al-Hallaq H Hoffman PC Jelinek MJ. A phase 1 trial of concurrent or sequential ipilimumab, nivolumab, and stereotactic body radiotherapy in patients with stage IV NSCLC study. J Thorac Oncol. (2022) 17:130–40. doi: 10.1016/j.jtho.2021.08.019 34500113

[8] VanderWalde A Bellasea SL Kendra KL Khushalani NI Campbell KM Scumpia PO. Ipilimumab with or without nivolumab in PD-1 or PD-L1 blockade refractory metastatic melanoma: a randomized phase 2 trial. Nat Med. (2023) 29:2278–85. doi: 10.1038/s41591-023-02498-y PMC10708907 37592104

[9] Liu K Yuan S Wang C Zhu H. Resistance to immune checkpoint inhibitors in gastric cancer. Front Pharmacol. (2023) 14:1285343. doi: 10.3389/fphar.2023.1285343 38026944 PMC10679741

[10] Yin Q Wu L Han L Zheng X Tong R Li L. Immune-related adverse events of immune checkpoint inhibitors: a review. Front Immunol. (2023) 14:1167975. doi: 10.3389/fimmu.2023.1167975 37304306 PMC10247998

[11] Jungles KM Holcomb EA Pearson AN Jungles KR Bishop CR Pierce LJ. Updates in combined approaches of radiotherapy and immune checkpoint inhibitors for the treatment of breast cancer. Front Oncol. (2022) 12:1022542. doi: 10.3389/fonc.2022.1022542 36387071 PMC9643771

[12] Blach-Olszewska Z Leszek J. Mechanisms of over-activated innate immune system regulation in autoimmune and neurodegenerative disorders. Neuropsychiatr Dis Treat. (2007) 3:365–72. PMC2654796 19300567

[13] Yang L Murthy S Cortellini A Lim EA Gonzalez M Pinato DJ. Effects of immune checkpoint inhibitor associated endocrinopathies on cancer survival. Front Endocrinol. (2024) 15:1369268. doi: 10.3389/fendo.2024.1369268 PMC11045886 38681767

[14] Pawelec G Picard E Bueno V Verschoor CP Ostrand-Rosenberg S. MDSCs, ageing and inflammageing. Cell Immunol. (2021) 362:104297. doi: 10.1016/j.cellimm.2021.104297 33550187

[15] Manguso RT Pope HW Zimmer MD Brown FD Yates KB Miller BC. *In vivo* CRISPR screening identifies Ptpn2 as a cancer immunotherapy target. Nature. (2017) 547:413–8. doi: 10.1038/nature23270 PMC5924693 28723893

[16] Borgeaud M Sandoval J Obeid M Banna G Michielin O Addeo A. Novel targets for immune-checkpoint inhibition in cancer. Cancer Treat Rev. (2023) 120. doi: 10.1016/j.ctrv.2023.102614 37603905

[17] Kallingal A Olszewski M Maciejewska N Brankiewicz W Baginski M. Cancer immune escape: the role of antigen presentation machinery. J Cancer Res Clin Oncol. (2023) 149:8131–41. doi: 10.1007/s00432-023-04737-8 PMC10374767 37031434

[18] Dawood ZS Brown ZJ Endo Y Katayama ES Munir MM Alaimo L. Cost effectiveness of immune checkpoint inhibitors for treatment of Hepatocellular Carcinoma: A systematic review and Meta-analysis. Surg Oncol. (2023) 51:102013. doi: 10.1016/j.suronc.2023.102013 39492244

[19] He R Yuan X Chen Z Zheng Y. Combined immunotherapy for metastatic triple-negative breast cancer based on PD-1/PD-L1 immune checkpoint blocking. Int Immunopharmacol. (2022) 113:109444. doi: 10.1016/j.intimp.2022.109444 36402069

[20] Hosseinkhani N Hemmat N Baghbani E Baghbanzadeh A Kazemi T Mokhtarzadeh A. Dual silencing of tumor-intrinsic VISTA and CTLA-4 stimulates T-cell mediated immune responses and inhibits MCF7 breast cancer development. Gene. (2024) 896:148043. doi: 10.1016/j.gene.2023.148043 38042220

[21] Ndjana Lessomo FY Wang Z Mukuka C. Comparative cardiotoxicity risk of pembrolizumab versus nivolumab in cancer patients undergoing immune checkpoint inhibitor therapy: A meta-analysis. Front Oncol. (2023) 13:1080998. doi: 10.3389/fonc.2023.1080998 37064101 PMC10090546

[22] Yolchuyeva S Giacomazzi E Tonneau M Lamaze F Orain M Coulombe F. Imaging-based biomarkers predict programmed death-ligand 1 and survival outcomes in advanced NSCLC treated with nivolumab and pembrolizumab: A multi-institutional study. JTO Clin Res Rep. (2023) 4:100602. doi: 10.1016/j.jtocrr.2023.100602 38124790 PMC10730368

[23] Tural D Arslan C Selcukbiricik F Olmez OF Erman M Ürün Y. Objective response rate is a surrogate marker for long-term overall survival in metastatic urothelial carcinoma patients treated with immune checkpoint inhibitors. Clin Genitourinary Cancer. (2024) 22:102163. doi: 10.1016/j.clgc.2024.102163 39126823

[24] Kirk R. CD8+:FOXP3+ cell ratio is a novel survival marker for colorectal cancer. Nat Rev Clin Oncol. (2010) 7:299–9. doi: 10.1038/nrclinonc.2010.79 20527684

[25] Zhao M Yan C-Y Wei Y-N Zhao X-H. Breaking the mold: Overcoming resistance to immune checkpoint inhibitors. Antiviral Res. (2023) 219:105720. doi: 10.1016/j.antiviral.2023.105720 37748652

[26] Tomm NK Szczepanski JM Fang JM Choi W-T Xue Y Setia N. Follow-up biopsies in gastrointestinal immune checkpoint inhibitor toxicity may show markedly different inflammatory patterns than initial injury. Hum Pathol. (2024) 148:60–5. doi: 10.1016/j.humpath.2024.05.001 38734079

[27] Wang SJ Dougan SK Dougan M. Immune mechanisms of toxicity from checkpoint inhibitors. Trends Cancer. (2023) 9:543–53. doi: 10.1016/j.trecan.2023.04.002 PMC10330206 37117135

[28] Ellis SR Vierra AT Millsop JW Lacouture ME Kiuru M. Dermatologic toxicities to immune checkpoint inhibitor therapy: A review of histopathologic features. J Am Acad Dermatol. (2020) 83:1130–43. doi: 10.1016/j.jaad.2020.04.105 PMC7492441 32360716

[29] Fazio R Audisio A Daprà V Conti C Benhima N Abbassi F-Z. Non-operative management after immune checkpoint inhibitors for early-stage, dMMR/MSI-H gastrointestinal cancers. Cancer Treat Rev. (2024) 128:102752. doi: 10.1016/j.ctrv.2024.102752 38772170

[30] Dong Y Zhi Y Mao Y. SAT-333 Efficacy of magnesium isoglycyrrhizinate as add-on therapy to glucocorticoids in immune checkpoint inhibitor-related hepatotoxicity. J Hepatol. (2024) 80:S122–3. doi: 10.1016/S0168-8278(24)00664-0

[31] Verheijden RJ Burgers FH Janssen JC Putker AE Veenstra SPGR Hospers GAP. Corticosteroids and other immunosuppressants for immune-related adverse events and checkpoint inhibitor effectiveness in melanoma. Eur J Cancer. (2024) 207:114172. doi: 10.1016/j.ejca.2024.114172 38905818

[32] Ancel J Dormoy V Raby BN Dalstein V Durlach A Dewolf M. Soluble biomarkers to predict clinical outcomes in non-small cell lung cancer treated by immune checkpoints inhibitors. Front Immunol. (2023) 14:1171649. doi: 10.3389/fimmu.2023.1171649 37283751 PMC10239865

[33] Zheng Y Fang YC Li J. PD−L1 expression levels on tumor cells affect their immunosuppressive activity. Oncol Lett. (2019) 18. doi: 10.3892/ol.2019.10903 PMC6781757 31612048

[34] Ding H Wang G Yu Z Sun H Wang L. Role of interferon-gamma (IFN-γ) and IFN-γ receptor 1/2 (IFNγR1/2) in regulation of immunity, infection, and cancer development: IFN-γ-dependent or independent pathway. Biomedicine Pharmacotherapy. (2022) 155:113683. doi: 10.1016/j.biopha.2022.113683 36095965

[35] Di Federico A Alden SL Smithy JW Ricciuti B Alessi JV Wang X. Intrapatient variation in PD-L1 expression and tumor mutational burden and the impact on outcomes to immune checkpoint inhibitor therapy in patients with non-small-cell lung cancer. Ann Oncol. (2024). doi: 10.1016/j.annonc.2024.06.014 38950679

[36] Ambrosini M Tougeron D Modest D Guimbaud R Kopetz S Decraecker M. BRAF + EGFR +/- MEK inhibitors after immune checkpoint inhibitors in BRAF V600E mutated and deficient mismatch repair or microsatellite instability high metastatic colorectal cancer. Eur J Cancer. (2024) 210:114290. doi: 10.1016/j.ejca.2024.114290 39216175

[37] Martinez-Morilla S Moutafi M Rimm DL. Standardization of PD-L1 immunohistochemistry. Modern Pathol. (2022) 35:294–5. doi: 10.1038/s41379-021-00917-4 PMC8860739 34508229

[38] Butler M Konigshofer Y Nguyen L Kalotra S Clement O Garlick R. Abstract 3167: Improving and standardizing TMB assay performance. (2019). doi: 10.1158/1538-7445.SABCS18-3167

[39] Yamamoto H Imai K. An updated review of microsatellite instability in the era of next-generation sequencing and precision medicine. Semin Oncol. (2019) 46:261–70. doi: 10.1053/j.seminoncol.2019.08.003 31537299

[40] Liang Y Wang L Ma P Ju D Zhao M Shi Y. Enhancing anti-tumor immune responses through combination therapies: epigenetic drugs and immune checkpoint inhibitors. Front Immunol. (2023) 14:1308264. doi: 10.3389/fimmu.2023.1308264 38077327 PMC10704038

[41] Zhang R Yu J Guo Z Jiang H Wang C. Camptothecin-based prodrug nanomedicines for cancer therapy. Nanoscale. (2023) 15:17658–97. doi: 10.1039/D3NR04147F 37909755

[42] Wang Z Ren X Wang D Guan L Li X Zhao Y. Novel strategies for tumor radiosensitization mediated by multifunctional gold-based nanomaterials. Biomaterials Sci. (2023) 11:1116–36. doi: 10.1039/D2BM01496C 36601661

[43] Butterfield LH Najjar YG. Immunotherapy combination approaches: mechanisms, biomarkers and clinical observations. Nat Rev Immunol. (2024) 24:399–416. doi: 10.1038/s41577-023-00973-8 38057451 PMC11460566

[44] Zhao H Yu J Zhang R Chen P Jiang H Yu W. Doxorubicin prodrug-based nanomedicines for the treatment of cancer. Eur J Medicinal Chem. (2023) 258:115612. doi: 10.1016/j.ejmech.2023.115612 37441851

[45] Jiang M Hu Y Lin G Chen C. Dosing regimens of immune checkpoint inhibitors: attempts at lower dose, less frequency, shorter course. Front Oncol. (2022) 12:906251. doi: 10.3389/fonc.2022.906251 35795044 PMC9251517

[46] Zhang R Zhao X Jia A Wang C Jiang H. Hyaluronic acid-based prodrug nanomedicines for enhanced tumor targeting and therapy: A review. Int J Biol Macromolecules. (2023) 249:125993. doi: 10.1016/j.ijbiomac.2023.125993 37506794

[47] Jiang H Gong Q Zhang R Yuan H. Tetrazine-based metal-organic frameworks. Coordination Chem Rev. (2024) 499:215501. doi: 10.1016/j.ccr.2023.215501

[48] Chen X Wu W Wei W Zou L. Immune checkpoint inhibitors in peripheral T-cell lymphoma. Front Pharmacol. (2022) 13:869488. doi: 10.3389/fphar.2022.869488 35559250 PMC9086454

[49] Jiang H Chen W Wang J Zhang R. Selective N-terminal modification of peptides and proteins: Recent progresses and applications. Chin Chem Lett. (2022) 33:80–8. doi: 10.1016/j.cclet.2021.06.011

[50] Wu X Feng N Wang C Jiang H Guo Z. Small molecule inhibitors as adjuvants in cancer immunotherapy: enhancing efficacy and overcoming resistance. Front Immunol. (2024) 15:1444452. doi: 10.3389/fimmu.2024.1444452 39161771 PMC11330769

[51] Tu Y Gong J Mou J Jiang H Zhao H Gao J. Strategies for the development of stimuli-responsive small molecule prodrugs for cancer treatment. Front Pharmacol. (2024) 15:1434137. doi: 10.3389/fphar.2024.1434137 39144632 PMC11322083

[52] Tang Q Chen Y Li X Long S Shi Y Yu Y. The role of PD-1/PD-L1 and application of immune-checkpoint inhibitors in human cancers. Front Immunol. (2022) 13:964442. doi: 10.3389/fimmu.2022.964442 36177034 PMC9513184

[53] Gault A Hogarth L Williams KC Greystoke A Rajan N Speight A. Monitoring immunE DysregulAtion foLLowing Immune checkpOint-inhibitioN (MEDALLION): protocol for an observational cancer immunotherapy cohort study. BMC Cancer. (2024) 24:733. doi: 10.1186/s12885-024-12468-3 38877461 PMC11179333

[54] Desmedt V Jauregui-Amezaga A Fierens L Aspeslagh S Dekervel J Wauters E. Position statement on the management of the immune checkpoint inhibitor-induced colitis via multidisciplinary modified Delphi consensus. Eur J Cancer. (2023) 187:36–57. doi: 10.1016/j.ejca.2023.03.025 37116287

[55] Marin-Acevedo JA Kimbrough EO Lou Y. Next generation of immune checkpoint inhibitors and beyond. J Hematol Oncol. (2021) 14:45. doi: 10.1186/s13045-021-01056-8 33741032 PMC7977302

[56] Ziogas DC Theocharopoulos C Koutouratsas T Haanen J Gogas H. Mechanisms of resistance to immune checkpoint inhibitors in melanoma: What we have to overcome? Cancer Treat Rev. (2023) 113:102499. 36542945 10.1016/j.ctrv.2022.102499

